# New strategy to elucidate the positive effects of extractable lignin on enzymatic hydrolysis by quartz crystal microbalance with dissipation

**DOI:** 10.1186/s13068-019-1402-2

**Published:** 2019-03-19

**Authors:** Chenhuan Lai, Bo Yang, Zihe Lin, Yuan Jia, Caoxing Huang, Xin Li, Xiangyang Song, Qiang Yong

**Affiliations:** 1 Jiangsu Co-Innovation Center of Efficient Processing and Utilization of Forest Resources, Nanjing Forestry University, Nanjing, 210037 China; 2 College of Chemical Engineering, Nanjing Forestry University, Nanjing, 210037 China

**Keywords:** Extractable lignin, Milled wood lignin, Enzymatic hydrolysis, Enzyme adsorption, Quartz crystal microbalance with dissipation

## Abstract

**Background:**

The presence of lignin normally affects enzymatic saccharification of lignocellulose detrimentally. However, positive effects of lignin on enzymatic hydrolysis have been recently reported. Enzyme–lignin interactions could be the key to reveal the underlying mechanism of their discrepant behaviors. In this study, to elucidate the positive effects of extractable lignin (EL) on enzymatic hydrolysis of ethanol organosolv-pretreated wood sawdust, two lignin fractions, EL and milled wood lignin (MWL), were isolated sequentially from pretreated substrates. Quartz crystal microbalance with dissipation (QCM-D) was then used to investigate the lignin aggregation effects on enzyme adsorption.

**Results:**

We found that both EL and MWL had a narrow molecular weight distribution. However, MWL had an obviously higher molecular weight than EL. This indicated that EL and MWL likely represent two distinct lignin fractions from ethanol organosolv-pretreated substrates. HSQC NMR analysis revealed that less β-*O*-4, β-β, and β-5 linkages and a higher S/G ratio was present in EL, as compared to MWL. QCM-D analysis showed that the enzyme adsorption on lignin was highly relevant to these lignin structural characteristics. An obviously lower maximum enzyme adsorption capacity was observed on EL films (152.63–168.09 ng/cm^2^) compared to MWL films (196.71–224.73 ng/cm^2^). Furthermore, enzyme desorption on lignin films was determined. A significantly lower irreversible enzyme adsorption was observed on EL (75.40 ng/cm^2^) compared to MWL (137.35 ng/cm^2^). More importantly, two reconstructed lignin films were designed to investigate lignin assembly on enzyme adsorption. The results indicated that the presence of EL reduced irreversible enzyme adsorption on the reconstructed lignin films by 39.2–45.0%.

**Conclusions:**

Lignin structure determined the interaction between enzyme and lignins. A positive correlation was observed between molecular weight, the content of β-5 linkages, and enzyme adsorption on lignin. EL, which was more depolymerized and less condensed, had the lower enzyme adsorption of the two preparations tested. Additionally, the presence of EL reduced enzyme adsorption on reconstructed lignin films, perhaps through a mechanism involving the blocking of non-productive enzyme binding sites on the MWL. This could be the mechanism for the positive effects of EL on enzymatic hydrolysis.

**Electronic supplementary material:**

The online version of this article (10.1186/s13068-019-1402-2) contains supplementary material, which is available to authorized users.

## Background

Lignocellulosic biorefineries have gained increasing research interest in the last decade due to the concerns about fossil resource depletion [1, 2]. In a typical biorefinery process, fermentable sugars are released from the biomass via enzymatic hydrolysis, and then the resultant sugars are fermented to valuable products [3–5]. Enzymatic hydrolysis has been considered to be one of the major economical bottlenecks during a biorefinery process [6]. Therefore, extensive efforts have been devoted to investigate the factors affecting enzymatic hydrolysis, such as inhibition from lignin, pseudo-lignin, or hemicellulose, cellulose accessibility, non-synergistic enzyme action, etc[7–12]. Among these influential factors, the inhibitory effects of lignin on enzymatic hydrolysis have been investigated extensively. It is agreed that lignin hinders enzymatic hydrolysis by physical blocking and enzyme non-productive binding [13]. A pretreatment with effective lignin removal is favored to relieve lignin inhibition, such as organosolv pretreatment or sulfite pretreatment [3]. However, part of lignin will still remain in pretreated substrates despite pretreatment, resulting in the enzyme non-productive binding [14].

To reduce the inhibitory effects of enzyme non-productive binding on enzymatic hydrolysis, the interaction mechanism between enzymes and lignin has been explored. The synergistic action of hydrophobic interactions, electrostatic interactions, and hydrogen bonding between enzymes and lignin has been suggested to determine the enzyme non-productive binding [15]. Moreover, the interaction between enzymes and lignin was associated with both of their physicochemical properties, such as hydrophobicity, magnitude of negative charge, and specific functional groups [16]. In the hydrophobic interactions between enzymes and lignin, the aromatic amino acid residues in cellulose-binding modules play an important role [16–18]. It has also been found that lignin with greater hydrophobicity typically enables stronger hydrophobic interactions between enzymes and lignin. Due to this phenomenon, the hydrophobicity of lignin surfaces has been experimentally lowered through the addition of surfactants, or by modifying lignin to imbue it with hydrophilic functionalities [19]. Both of the aforementioned modifications turned out to be capable strategies for reducing non-productive enzyme binding and improving enzymatic digestibility of biomass. As for electrostatic interactions, the association or dissociation of functional groups in enzymes and lignin appears to be the dominating factor (e.g., carboxyl and hydroxyl groups) [16]. Normally, cellulases from *Trichoderma reesei* are mostly negatively charged at the suggested enzymatic hydrolysis pH (4.8). Thus, pretreatments that introduce negatively charged groups to the lignins, together with the elevation of the hydrolysis pH from 4.8 to 5.2–6.0, have been employed to enhance electrostatic repulsion between enzymes and lignin. This enhanced repulsion also lead to a decline in enzyme non-productive bindings and improved enzymatic hydrolysis efficiency [20, 21]. In addition, both phenolic hydroxyl groups and the condensed subunits in lignin have been reported to increase the enzymatic affinity toward lignin [22–25]. The former might increase the formation of hydrogen bonding between enzymes and lignin [22], while the latter has been suggested to enhance hydrophobic interactions between the two [24]. Therefore, chemically blocking lignin phenolic hydroxyl groups and suppressing lignin condensation during pretreatment have been proposed to alleviate enzyme non-productive binding [22, 26, 27].

Extensive efforts have been devoted to investigate the negative effects of lignin on enzymatic hydrolysis, and how to relieve its inhibition. However, the positive effects of lignin on enzymatic hydrolysis have also been reported upon recently [21, 28]. Lignosulfonate has been observed to form lignosulfonate–cellulase complexes, which are believed to enhance electrostatic repulsion between the enzyme and residual bulk lignin. This interaction was shown to improve enzymatic hydrolysis of lignocellulose [21, 29]. Our own previous work observed that lignin obtained from ethanol organosolv-pretreated sweetgum (“EL”) surprisingly showed a positive effect on enzymatic hydrolysis [28]. However, the underlying mechanism for the positive effects of EL was not fully investigated. In an attempt to reveal the potential mechanism, both EL and the residual bulk lignin were sequentially fractionated from ethanol organosolv-pretreated mixed wood sawdust. EL was simply isolated from the pretreated substrates by ethanol extraction due to its favorable ethanol solubility. Milled wood lignin (“MWL”) was then isolated from the ethanol extracted residues to represent the residual bulk lignin to serve as a representative isolate of the residual lignin [24]. Both isolated lignin fractions’ chemical structures were elucidated by nuclear magnetic resonance (NMR) analysis. In addition, enzyme adsorption on these two lignin fractions was monitored by QCM-D analysis in real time. Furthermore, the effects of the assembly between EL and MWL on the enzyme adsorption were revealed by QCM-D analysis. The results herein will aid in developing a stronger collective understanding of the positive and negative effects exerted by lignin upon enzymatic hydrolysis.

## Methods

### Ethanol organosolv pretreatment of mixed wood sawdust

Mixed wood sawdust [0.5 × 0.5 cm (*L* × *W*)] primarily composed of softwood was collected from Xuzhou, Jiangsu province, China, and used as the raw material for the ethanol organosolv pretreatment experiments. Pretreatment was carried out in a rotary cooking system (YRG2-10 × 1.25, Nanjing Jiezheng, Jiangsu, China) with ten 1.25-L stainless-steel bomb reactors and an electrically heated oil bath. Prior to pretreatment, wood sawdust (80 g, dry weight) was soaked overnight at room temperature in 25% or 50% (v/v) ethanol solution with 1% sulfuric acid (based on biomass) at a solid–liquid ratio of 1:10. After soaking, the wood sawdust and cooking liquor were transferred into the bomb reactor and pretreated at 180 °C for 60 min. The pretreatment conditions were chosen according to our previous study [28]. After pretreatment, the bomb reactors were cooled in an ice water bath. Neither the heating time (about 30 min) to reach 180 °C, nor the cooling time (about 15 min) was included in the pretreatment time (60 min). The pretreated slurry was separated into a solid fraction and a liquid fraction by filtration. The solid substrate was then washed extensively with tap water until the wash filtrate reached neutral pH, and then collected by filtration. The obtained solid substrate pretreated with 25% ethanol was termed as EP25, while the material pretreated with 50% ethanol was designated as EP50.

### Ethanol washing on ethanol organosolv-pretreated substrates

To remove EL, ethanol washing was carried out upon the ethanol organosolv-pretreated substrates (EP25 and EP50) according to the methodology of our previous study [28]. Briefly, the pretreated substrates were mixed with 95% ethanol at a solid–liquid ratio of 1:10 at room temperature for 5 min. After that, the pretreated solids were collected by filtration. This ethanol-washing process was repeated three times. After three washes, the collected substrates were further washed with tap water. The resultant triply washed substrates were referred to EP25-EW and EP50-EW, respectively. The filtrate from the ethanol washing was also collected for the later EL preparation.

### Chemical composition analysis of pretreated substrates

The chemical compositions of pretreated substrates were analyzed according to the National Renewable Energy Laboratory protocol [30]. First, the quantity of ethanol extractive was gravimetrically measured by ethanol extraction. Next, extractive-free samples were hydrolyzed by two-step sulfuric acid hydrolysis (72% sulfuric acid at 30 °C for 1 h, and followed by 4% sulfuric acid at 121 °C for 1 h). Differently, the lignin samples (MWL and EL) were directly subjected to the two-step sulfuric acid hydrolysis without ethanol extraction. The concentrations of glucose released during acid hydrolysis were analyzed by high-performance liquid chromatography (HPLC, Agilent 1260, Palo Alta, CA, USA) with Aminex HPX-87P column (Bio-Rad, Hercules, CA, USA), and used to calculate the glucan content of the substrates. The concentrations of mannose, xylose, and arabinose were also determined using the same HPLC setup to calculate the hemicellulose contents in the substrates. Acid-insoluble lignin contents were determined by weighing the solid residues remaining after acid hydrolysis.

### Scanning electron microscopy (SEM)

The lignocellulosic material samples were coated with a very thin gold layer using a sputter coater prior to the SEM analysis. After coating, SEM analysis was performed with a field emission SEM (JEOL-JSM 7600F, JEOL, Tokyo, Japan).

### Cellulose accessibility determination by direct red dye adsorption

Cellulose accessibility of the raw and pretreated materials (EP25, EP25-EW, EP50, and EP50-EW) was determined by direct red dye (DR28) adsorption assays [31]. The direct red adsorption assay was carried out at 1% (w/v) lignocellulosic material suspended with a series of increasing direct red dye concentrations (0.00, 0.05, 0.10, 1.00, 2.00, 3.00, and 4.00 g/L), which was then incubated at 50 °C and 150 rpm for 24 h. After that, the samples were taken and centrifuged at 10,000 rpm for 5 min. The absorbance of the supernatants from samples was measured at 498 nm to determine the concentrations of direct red dye in the supernatant. The amount of adsorbed dye was then calculated as the difference between the dye concentrations in the supernatant and the initial dye concentrations. Langmuir non-linear regression was used to determine the maximum adsorption capacity of direct red dye, which served as an estimate of cellulose accessibility to enzymes within each tested substrate [31, 32].

### Enzymes

Commercial cellulase (UTA-8) was provided by Youtell Biochemical Co., Ltd. (Hunan province, China). Its filter paper activity, β-glucosidase activity, and protein content were 70.6 FPIU/mL, 27.5 IU/mL, and 29.9 mg/mL, respectively. Commercial β-glucosidase (BG188) with β-glucosidase activity of 205.4 IU/mL, and protein content of 53.8 mg/mL was provided by Novozymes (Beijing, China). Cellulase (UTA-8) was supplemented with β-glucosidase (BG188) to reach a filter paper activity to β-glucosidase activity ratio of 1:1. This enzyme blend with protein content of 34.0 mg/mL was applied in the following enzymatic hydrolysis experiments.

### Enzymatic hydrolysis of ethanol organosolv-pretreated substrates

Enzymatic hydrolysis was carried out in sodium citrate buffer (pH 4.8) at 20 g/L glucan with the aforementioned enzyme blend at 50 °C and 150 rpm for 72 h. An enzyme loading of 9.6-mg protein per g glucan in the pretreated solids (20 FPIU/g glucan) was used. Aliquots were withdrawn from the hydrolysis suspension at selected time intervals (3, 6, 12, 24, 48, and 72 h) and centrifuged at 10,000 rpm for 10 min. Monosaccharide concentrations were measured in each withdrawn sample’s supernatant using the previously described HPLC system. Hydrolysis yields were calculated based on the released glucose quantities as a percentage of the theoretical glucose available in the substrates. The corresponding calculation formula is as follows:

$$ {\text{Hydrolysis yield (}}\% ) { = }\frac{\text{Glucose in enzymatic hydrolyzate (g/L)}}{{ 1. 1 0\times {\text{glucan in pretreated solids (g/L)}}}} \, \times { 100}\% $$

### Fractionation of lignin from ethanol organosolv-pretreated substrates

**Fig. 1:**
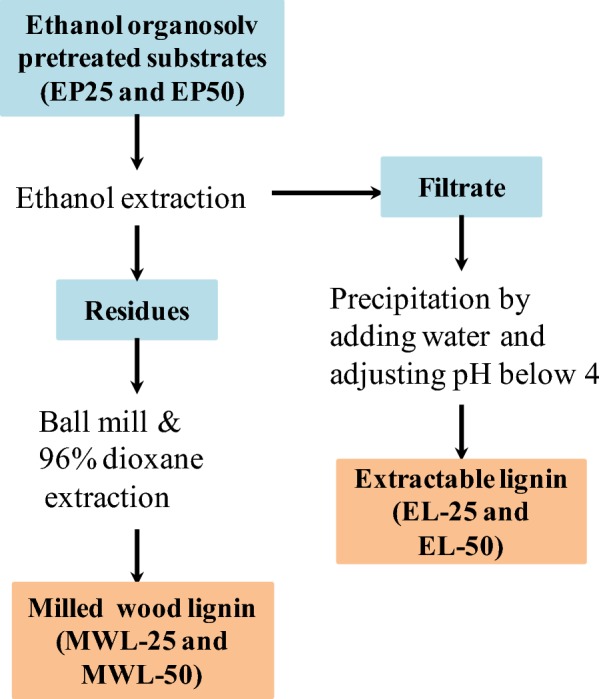
Fractionation of lignins from ethanol organosolv-pretreated substrates

Two lignin fractions (EL and MWL) were sequentially isolated from the pretreated substrates (EP25 and EP50) as shown in Fig. 1. First, the pretreated substrates were extracted extensively using ethanol. The ethanol extraction solution and the extracted solid residues were then separated by filtration. Next, EL was precipitated from the ethanol extraction solution by addition of threefold volume of water and then adjusting the pH below 4 with dilute sulfuric acid. The precipitated EL lignin was collected by filtration, washed with distilled water at least three times, and finally freeze dried. The obtained EL from EP25 and EP50 is referred to as EL-25 and EL-50, respectively.

After ethanol extraction, there was still residual bulk lignin remaining in the ethanol-extracted solid residues due to both its tight association with polysaccharides and poor solubility in ethanol. To isolate this residual bulk lignin, an MWL preparation was procured using the classical MWL isolation method [33]. The EL-free biomass was subjected to ball milling for 6 h. The resultant ball-milled fine powder was then suspended in 1,4-dioxane/water (96:4, v/v) at a solid–liquid ratio of 1:20, and lignin extraction was performed at room temperature for 24 h in the dark. After that, the solid residue and the extract solution were separated by filtration. To ensure theoretical maximum extraction, the captured solid residue was again extracted with fresh solvent two more times. The obtained solvent filtrates, the additional extraction stages were then combined and concentrated with a rotary evaporator to remove dioxane. The resultant crude lignin sample went through further purification according to a reported method, and was finally freeze-dried [34]. Finally, the isolated MWL lignin from ethanol extractive-free EP25 and EP50 was obtained and is referred to MWL-25 and MWL-50, respectively.

### Lignin characterization (GPC, HSQC NMR, and ^31^P NMR)

Molecular weight determination was performed on EL and MWL using gel permeation chromatography (GPC). Acetylation of each lignin preparation was performed prior to analysis [22]. GPC analysis took place within an HPLC system (Agilent 1200, Palo Alta, CA, USA) equipped with three Styragel columns (HR5E, HR4, and HR2) in tandem, and a refractive index detector. The acetylated lignins (1 mg) were dissolved in 1 mL of HPLC-grade tetrahydrofuran (THF), and 50 μL of sample was injected into the system. GPC calibration was performed with commercial polystyrene standards.

HSQC NMR analysis was also carried out on lignin samples using a Bruker AVANCE 600 MHz spectrometer (Bruker Biospin, Billerica, MA, USA) with the pulse program “hsqcetgp” as described previously [24]. EL and MWL (60 mg) were dissolved in 0.5 mL of deuterated dimethyl sulfoxide (DMSO-d_6_). The analysis and processing parameters were as follows: the number of collected complex points was 1 K for the ^1^H dimension with d_1_ (2 s), the number of scans was 64, and 256 time increments were always recorded.

Quantitative ^31^P NMR spectra of the lignin preparations were also acquired by a Bruker AVANCE 600 MHz spectrometer. First, an accurately weighed amount (40 mg) of dried samples was added into an NMR tube with 500-μL mixture of anhydrous pyridine and CDCl_3_ (1.6:1, v/v). Once the samples dissolved, 200 μL of endo-*N*-hydroxy-5-norbornene-2, 3-dicarboximide (e-NHI) solution (9.23 mg/mL) was added as an internal standard, as well as 50 μL of chromium (III) acetylacetonate solution (5.6 mg/mL) to serve as relaxation reagent. Finally, 100 μL of 2-chloro-4, 4, 5, 5-tetramethyl-1, 2, 3-dioxaphospholane (TMDP, a phosphitylating reagent) was added to the NMR tube which was then inverted several times for mixing. After derivatization, the prepared sample was immediately subjected to ^31^P NMR analysis.

### Lignin film preparation

Lignin films were prepared on gold-coated QCM sensors (QSX301, Västra Frölunda, Sweden) as previously described [35]. Prior to film preparation, QCM gold sensors were cleaned via treatment with 25% ammonia solution/30% hydrogen peroxide/water (1:1:5, v/v/v) at 75 °C for 5 min, rinsed with deionized water, and finally dried under nitrogen. After cleaning, 0.5% (w/v) lignin solutions were prepared by completely dissolving four lignin samples (MWL-25, EL-25, MWL-50, and EL-50) in DMSO, respectively. Then, the lignin solution was coated on QCM gold sensors with a spin coater (WS650MZ23NPPB, Laurell, USA) operating at 5000 rpm for 1 min. The spin coating process was repeated twice. The films were vacuum dried at 40 °C for 4 h to remove the bulk of the remaining DMSO. After that, the lignin films were soaked in deionized water until all DMSO was removed, and vacuum dried again prior to experimentation. Finally, the four lignin films of MWL-25, EL-25, MWL-50, and EL-50 were obtained.

To investigate the effects of EL on enzyme adsorption on MWL, two types of reconstructed lignin films were prepared. One type reconstructed lignin film was prepared using a 0.5% lignin solution with MWL-25 and EL-25 mixed in a ratio of 1:1. This sample is referred to EL/MWL-25 film. Another reconstructed lignin film was prepared by coating EL-25 on MWL-25 film, assigned as EL-MWL-25 film.

### Characterization of lignin films

The morphology and roughness of the lignin films were characterized by atomic force microscopy (AFM) (Dimesion Edge, Brucker, Saarbrücken, Germany). The images were scanned in tapping mode, and analyzed using NanoScope analysis software according to a previous report [35].

### Enzyme adsorption on lignin films determined by QCM-D

Enzyme adsorption on the four different lignin films (MWL-25, EL-25, MWL-50, and EL-50) was monitored using a quartz crystal microbalance (QCM-D E4 model, Biolin Corp., Gothenburg, Sweden). Prior to analysis, sodium citrate buffer (pH 4.8) was introduced into the measuring chambers by a peristaltic pump at a flow rate of 0.1 mL/min. Once a stable signal was reached, cellulase solution (protein concentration 0.1 mg/mL) was injected at a same flow rate of 0.1 mL/min. After adsorption equilibrium was reached, the QCM frequency changes (∆*f*, Hz) were fitted with Lagergren kinetic equation (*Δf* = *M*_max_(1 − *e*^−*t*/*τ*^)), where *M*_max_ (Hz) was referred as the maximum frequency changes, *t* (min) was time, and *τ* (min^−1^) was the binding rate [36]. The maximum adsorption capacity could then be calculated according to Sauerbrey equation ($\Delta m = - C\frac{\Delta f}{n}$, *n* = 3, *C* = 17.7 ng cm^−2^ Hz^−1^) [37].

To determine the effects of EL on enzyme adsorption on residual bulk lignin, the enzyme adsorptions on four lignin films (MWL-25, EL-25, EL/MWL-25, and EL-MWL-25) were compared. The maximum adsorption capacity was determined according to the method mentioned above. To distinguish the reversible and irreversible adsorption, the enzyme-free buffer was injected as a rinse after the enzyme adsorption equilibrium was reached on MWL-25, EL-25, EL/MWL-25, and EL-MWL-25. Frequency changes (∆*f*) for the fundamental frequency (5.0 MHz) were recorded as well as its overtones (*n* = 3, 5, 7, 9, 11, and 13). However, only the third overtone (*n* = 3) was used in the data evaluation. The temperature was maintained at 50 °C in all experiments, and each condition was tested at least three times [38].

## Results and discussion

### Chemical compositions of ethanol organosolv-pretreated wood sawdust

Ethanol organosolv pretreatment was performed at two different ethanol concentrations using wood sawdust as the pretreatment substrate. The chemical compositions of the raw and pretreated materials are shown in Table 1. Meanwhile, the recovery and removal of each component in pretreated materials are calculated by mass balance based on 100-g dry raw material (Additional file 1: Table S1). Results showed that pretreatment removed significant quantities of hemicellulose. In addition (and as intended), lignin was also partly removed. It was observed that the higher ethanol concentration we applied resulted in greater levels of delignification. Also as expected, removal of both hemicellulose and lignin resulted in a remarkable increase of the glucan contents from the original 42.6% in the starting material to 62.1% in EP25 and 66.6% in EP50, respectively.

**Table 1:** Chemical composition/cellulose accessibility of ethanol-pretreated biomass

Biomass	Extractives (%)	AIL^a^ (%)	Glucan (%)	Hemicellulose (%)	*Γ*_max_/DR28^b^ (mg/g)
Raw material	1.34 ± 0.11	28.22 ± 0.20	42.59 ± 0.98	22.70 ± 0.42	82.10
EP25	8.04 ± 0.60	26.41 ± 0.24	62.13 ± 0.75	6.93 ± 0.11	348.15
EP25-EW	0.94 ± 0.40	27.57 ± 0.74	65.67 ± 0.81	7.33 ± 0.03	368.52
EP50	7.70 ± 0.12	20.14 ± 2.53	66.56 ± 0.06	9.19 ± 0.04	382.84
EP50-EW	0.77 ± 0.04	18.94 ± 0.45	71.92 ± 0.26	9.67 ± 0.06	406.69

^a^AIL refers to the acid-insoluble lignin

^b^*Γ*_max_/DR28 refers to the maximum adsorption capacity of direct red dye (DR28) on pretreated materials, interpreted as cellulose accessibility

Another interesting observation was that the content of ethanol extractives increased significantly from 1.3% in the original material to 8.0% in EP25 and 7.7% in EP50. The mass balance (based on 100 g dry raw material) result also showed that the content of ethanol extractives increased obviously from 1.34 g in the raw material to 4.64 g in EP25 and 4.22 g in EP50, respectively (Additional file 1: Table S1). This increase in ethanol extractives can be mostly attributed to re-deposition of dissolved lignin upon the surfaces of the pretreated substrates [28]. This was verified by the SEM images of EP25 and EP50, in which lignin droplets were observed on the surfaces of biomass samples (Additional file 2: Figure S1). The temperature drop at the end of the pretreatment obviously reduced the solubility of dissolved lignin in the pretreatment liquor, leading to its precipitation. Moreover, the water addition further reduced the lignin solubility, and facilitated lignin re-deposition during the extensive water washing process of the pretreated residues. Due to its good ethanol extractability, this redeposited lignin has been defined as extractable lignin (EL), and the contents of EL were determined by ethanol extraction in our previous study [28]. It should be noted that the droplets could be also composed of pseudo-lignin and re-deposited hemicellulose [11, 39]. However, it is difficult to distinguish pseudo-lignin from real lignin. Furthermore, content of the re-deposited hemicellulose could be negligible compared to lignin content. The details of droplets compositions still need to be investigated in the future work.

To examine the effects of EL on enzymatic hydrolysis, ethanol washing was performed to remove EL from EP25 and EP50. The chemical compositions of the corresponding ethanol-washed biomass (EP25-EW and EP50-EW) are also shown in Table 1. Results showed that the ethanol washing efficiently reduced the contents of ethanol extractives to 0.9% in EP25-EW and 0.8% in EP50-EW. It was confirmed by SEM analysis that the lignin droplets were hardly observable on EP25-EW and EP50-EW compared to the corresponding un-extracted materials (Additional file 2: Figure S1). The mass balance result also showed that the ethanol washing efficiently removed ethanol extractives from EP25 by 89.0%, while 1.3% of acid-insoluble lignin and nearly none of glucan and hemicellulose were removed from EP25. Similarly, 90.8% of ethanol extractives were removed by the ethanol washing from EP50, but only a small amount of acid-insoluble lignin, glucan, and hemicellulose was removed by the ethanol washing from EP50 (Additional file 1: Table S1). These indicated that the main component removed by the ethanol washing was ethanol extractives, which was mostly composed of EL. Due to the removal of ethanol extractives, the contents of other components (such as acid-insoluble lignin, hemicellulose, and glucan) increased correspondingly in the ethanol-washed substrates (EP25-EW and EP50-EW).

### Effects of extractable lignin removal on cellulose accessibility and enzymatic digestibility of ethanol organosolv-pretreated wood sawdust

Cellulose accessibility has been considered as a key predictor of enzymatic digestibility [40], and it has been successfully estimated using the maximum adsorption capacity (*Γ*_max_) of DR28 dye on lignocellulosic materials [31]. Therefore, *Γ*_max_ values of DR28 on the four pretreated substrates were determined (Table 1). With EL removal, the *Γ*_max_ value of DR28 increased from 348.15 mg/g in EP25 to 368.52 mg/g in EP25-EW, and from 382.84 mg/g in EP50 to 406.69 mg/g in EP50-EW. These findings indicate that EL removal increased cellulose accessibility in both samples tested. This could be due to the fact that the re-deposition of EL is prevented when this material is extracted, preventing it from blocking access of enzymes to cellulose [41].

**Fig. 2:**
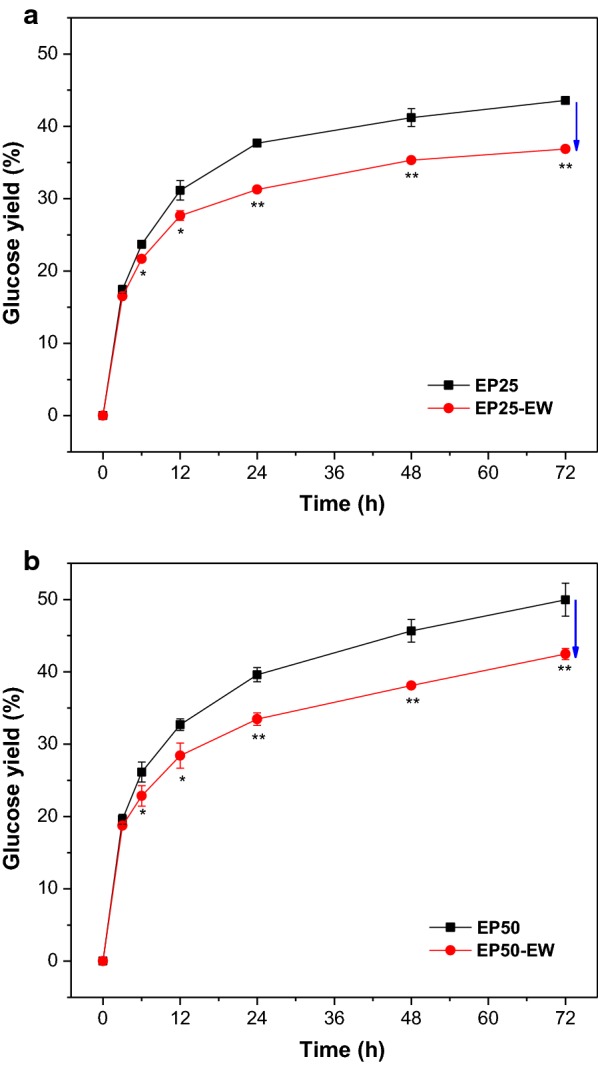
Effects of extractable lignin removal on enzymatic hydrolysis of **a** EP25, and **b** EP50. *p < 0.05, **p < 0.01 vs EP25 and EP50

To evaluate the effects of EL removal on the enzymatic digestibility, enzymatic hydrolysis was performed on each of the four pretreated substrates (Fig. 2). The results showed that EL removal decreased the 72-h hydrolysis yields from 43.6% (EP25) to 36.9% (EP25-EW), and from 50.0% (EP50) to 42.5% (EP50-EW). This suggests that EL removal decreased the enzymatic hydrolysis efficiency of EP25 and EP50. A similar result has been reported in the investigation of the EL effects on enzymatic hydrolysis of organosolv-pretreated sweetgum and dilute acid-pretreated sweetgum [28]. Normally, the increase of cellulose accessibility would lead to the improvement on enzymatic hydrolysis [40]. However, our results showed that EP25-EW and EP50-EW with the higher cellulose accessibility exhibited poorer enzymatic digestibility. The previous study observed higher enzyme adsorption on ethanol washed substrates, implying that the presence of EL might reduce the enzyme non-productive binding on the residual bulk lignin [28]. Therefore, it seems that when the cellulose accessibility of pretreated substrates was similar, the enzyme non-productive binding played a more important role in inhibiting enzymatic hydrolysis [42]. Furthermore, the negative effects of EL on reducing cellulose accessibility might be counteracted by the positive effects of EL on reducing enzyme non-productive binding. To verify this hypothesis, the EL and residual bulk lignin were fractionated from the pretreated substrates to investigate enzyme adsorption on both EL and residual bulk lignin.

### Characterization of lignin fractions

Lignin’s effects on enzyme adsorption and enzymatic hydrolysis could be related to its chemical structure. To probe this conjecture, EL and residual bulk lignins were isolated from the pretreated substrates (EP25 and EP50) and their chemical structures were analyzed. EL was precipitated from the ethanol washing filtrate, while a MWL preparation was isolated from the extractive-free pretreated substrates. The contents of acid-insoluble lignin were between 89.5 and 95.7% in EL and MWL (Table 2), while the contents of residual carbohydrate were only 0.8–2.5%. These two factors combined indicate that the lignin isolates are highly pure lignin preparations.

**Table 2:** Chemical composition/molecular weights of lignin fractions from ethanol organosolv-pretreated wood sawdust

Lignin	Chemical composition (%)			Molecular weight		
	AIL	Glucan	Hemicellulose	*M*_w_	*M*_n_	*M*_w_/*M*_n_
MWL-25	95.50 ± 0.17	0.62 ± 0.10	0.23 ± 0.01	6969	4932	1.41
MWL- 50	95.67 ± 0.67	0.62 ± 0.07	0.33 ± 0.13	8332	5028	1.66
EL-25	89.50 ± 2.16	1.50 ± 0.09	0.95 ± 0.02	1665	1240	1.34
EL-50	90.00 ± 2.00	1.21 ± 0.01	1.12 ± 0.12	1902	1330	1.43

^a^AIL refers to the acid-insoluble lignin

First, molecular weight analysis was performed to reveal the differences in the molecular weight distribution between the EL and MWL (Table 2). Analysis showed that the molecular weight of EL (*M*_w_= 1665–1902) was much lower than that of MWL (*M*_w_= 6969–8332) in both EP25 and EP50. This implied that lignin depolymerization helped to produce the EL fraction, resulting in its ethanol solubility. The residual bulk lignin, which is decidedly not ethanol soluble and is represented by MWL, has a relatively high molecular weight. This elevated molecular weight likely contributed to its poor ethanol solubility. Moreover, lignin depolymerization and repolymerization may have happened simultaneously during the pretreatment [26]. This dual occurrence potentially generated two lignin fractions with different molecular weights after pretreatment, both of which are analyzed in this work through the EL and MWL preparations. The similar phenomenon has been reported that the lignin extracted from steam-exploded aspen, which had GPC weight distribution curves with two distinct peaks indicating varied molecular weight fractions [18]. Finally, the degrees of polydispersity (*M*_w_/*M*_n_ < 2.0) suggest that both of the isolated lignin fractions have a narrow molecular weight distribution.

In addition, the molecular weight was observed lower in lignin fractions (MWL-25 and EL-25) isolated from EP25, compared to MWL-50 and EL-50. The similar phenomenon has been reported by Pan et al. that the molecular weight of ethanol organosolv lignin decreased linearly with decreasing ethanol concentration [43]. This could be due to the solvent effects on organosolv pretreatment. Our previous study observed that pH value was lower in ethanol organosolv pretreatment liquor with the same H_2_SO_4_ concentration but the lower ethanol concentration [44]. The decrease in ethanol concentration could reduce the pH by increasing the activity coefficient in the solution. Therefore, the stronger lignin degradation occurred during ethanol organosolv pretreatment with 25% (v/v) ethanol, as compared to that with 50% (v/v) ethanol, due to the lower pH in 25% (v/v) ethanol solution. This could be used to explain the lower molecular weight of lignins from EP25.

HSQC NMR analysis was carried out to detail the chemical structures of EL and MWL (Table 3). In the aliphatic side chain region (*δ*_C_/*δ*_H_ 50–90/2.5–6.0), the inter-unit linkages of β-aryl-ether (β-*O*-4, A), resinol (β-β, B), and phenylcoumaran (β-5, C) could be identified. The number of the inter-unit linkages per 100 aromatic units (Ar) was quantified using the aromatic units as internal standard [45]. Quantification found the contents of β-*O*-4 linkages to be 13.70–14.46 per 100 Ar in MWL, and 3.22–3.23 per 100 Ar in EL. As compared to the original lignin from raw material (~ 50 per 100 Ar) [46], the amount of β-*O*-4 linkages significantly decreased after the ethanol organosolv pretreatment. This reduction indicates that considerable cleavage of β-*O*-4 linkages took place during pretreatment [24, 45]. Both β-β and β-5 linkages, considered to be condensed subunits in lignins, were also quantified. Compared to MWL, lower abundancies of β-*O*-4, β-β, and β-5 linkages were observed in EL. This further demonstrates that EL was more significantly depolymerized and less condensed. These findings are consistent with the molecular weight analysis and its corresponding conjecture (Table 2). HSQC NMR analysis also allows for separation of syringyl (S) and guaiacyl (G) unit signals in the aromatic region (*δ*_C_/*δ*_H_ 100–130/6.0–8.0). The S/G ratios calculated from these signals showed that the S/G ratios were higher in EL (0.09–0.12), but were lower in MWL (0.03–0.05). This difference could be explained by the suggestion that β-*O*-4 linkages were more readily cleaved in lignin with more S units, thus leading to the lower molecular weight and better ethanol extractability of EL [47].

**Table 3:** Quantitative analysis of lignin substructures by 2D HSQC NMR

Lignin	S/G	β-*O*-4^a^	β-β^a^	β-5^a^
MWL-25	0.05	14.46	2.16	7.00
MWL-50	0.03	13.70	2.10	7.09
EL-25	0.12	3.22	1.51	4.37
EL-50	0.09	3.23	0.41	3.66

^a^Amount of inter-unit linkages was expressed as per 100 Ar

To address the presence of functional groups, the hydroxyl groups in MWL and EL were quantified using a ^31^P NMR technique (Table 4). Our findings showed that both EL preparations had a lower content of aliphatic hydroxyl groups compared to MWL lignins. This reduction could be attributable to the occurrence of elimination reactions or α-ethoxylation occurring more frequently on the aliphatic hydroxyl groups of EL [45]. Furthermore, greater quantities of phenolic hydroxyl groups were observed in EL (2.58–2.76 mmol/g) compared to MWL (1.85–2.04 mmol/g). This was likely due to a more significant cleavage of β-*O*-4 linkages in the EL preparations, something which was confirmed by the previously discussed HSQC NMR analysis (Table 3). Finally, quantities of carboxylic acid groups were lower in EL than MWL. This may be due to a potential esterification reaction occurring at these functionalities during the ethanol organosolv pretreatment [45].

**Table 4:** ^31^P NMR quantitative analysis of lignin fractions from ethanol organosolv-pretreated wood sawdust (mmol/g lignin)

Lignin	Aliphatic OH	Phenolic OH			Carboxylic OH
		Condensed	Non-condensed	Total	
MWL-25	2.80	0.77	1.27	2.04	0.75
MWL-50	3.32	0.61	1.23	1.85	0.36
EL-25	1.78	1.03	1.73	2.76	0.42
EL-50	2.10	0.92	1.66	2.58	0.30

### Enzyme adsorption on lignin films determined by QCM-D

To evaluate the adsorption affinity between enzymes and each of the two different lignin fractions, QCM-D analysis was applied to monitor the enzyme adsorption on generated MWL and EL films. Prior to the QCM-D analysis, films of MWL-25, EL-25, MWL-50, and EL-50 were prepared, their topographic images were scanned by AFM, and the corresponding root-mean-square (RMS) roughness was calculated. These calculations revealed that RMS roughness of each sample was in the range of 0.94–1.57 nm, indicating that the lignin films were sufficiently smooth for QCM-D analysis. Next, dynamic enzyme adsorption on the films of MWL-25, EL-25, MWL-50, and EL-50 were monitored using QCM-D. As enzyme adsorption equilibrium was reached, the QCM frequency changed during enzyme adsorption on four lignin films. These frequency changes could be fitted with the Lagergren kinetic equation to calculate maximum frequency changes (*M*_max_) (Table 5). Such calculations showed that the absolute values of *M*_max_ were 38.09, 25.87, 33.34, and 28.07 Hz for MWL-25, EL-25, MWL-50, and EL-50 films, respectively. Translating this information into the Sauerbrey equation [37], it was also found that the maximum adsorption capacity was calculated as 224.73, 152.63, 196.71, and 168.09 ng/cm^2^ for MWL-25, EL-25, MWL-50, and EL-50 films, respectively. These values indicate that the MWL films have an obviously higher enzyme adsorption capacity, potentially leading to the stronger inhibitory effects of residual lignin during enzymatic hydrolysis, as compared to EL.

**Table 5:** Enzyme adsorption parameters on lignin films as measured by QCM-D

Lignins	− *M*_max_ (Hz)	Maximum adsorption capacity (ng/cm^2^)	irreversible adsorption mass (ng/cm^2^)
MWL-25	38.09	224.73	137.35
EL-25	25.87	152.63	75.40
MWL-50	33.34	196.71	–
EL-50	28.49	168.09	–
EL/MWL-25^a^	28.07	165.61	83.54
EL-MWL-25^b^	24.11	142.25	75.58

^a^EL/MWL-25 refers to the lignin film prepared using a 0.5% lignin solution with MWL-25 and EL-25 mixed in a ratio of 1:1

^b^EL-MWL-25 refers to the lignin film prepared by coating EL-25 on MWL-25 film

**Fig. 3:**
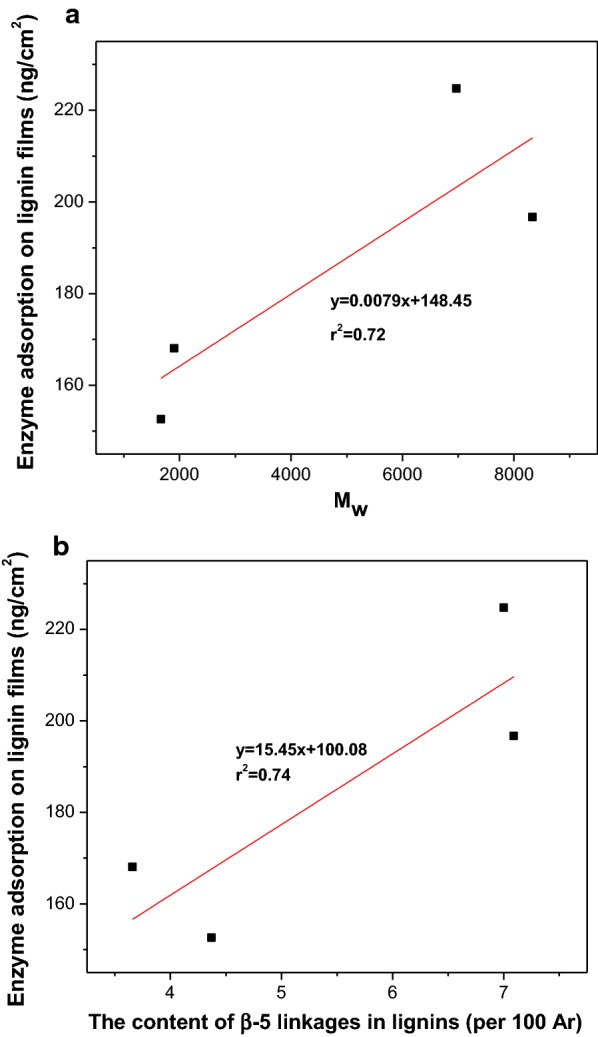
Correlation between lignin structural characteristic and enzyme adsorption on lignin films

The differences in enzyme adsorption capacity between lignin preparations were mainly related to structural characteristics that varied between each preparation. A positive correlation was observed between molecular weight (*M*_w_) and enzyme adsorption on lignin films (*r*^2^ = 0.72, Fig. 3a). A similar conclusion has been reported in other works, which stated that lignin with lower molecular weight may have lesser inhibitory effects upon enzymatic conversion [48]. Moreover, a positive correlation was also observed between the content of β-5 linkages and enzyme adsorption (*r*^2^ = 0.74, Fig. 3b). This was probably due to the stronger hydrophobic interaction between enzymes and lignin containing these subunits [24]. However, unlike the previous study [21], higher quantities of carboxylic acid groups in MWL did not correlate with lower enzyme adsorption (Table 4). Similarly, the more phenolic hydroxyl groups present in EL did not lead to them experienced more enzyme adsorption. This suggests that the hydrophobic, electrostatic, and hydrogen-bonding interactions might compete or collaborate with each other to result in a net effect upon enzyme adsorption [48], and the lignin structural features synergistically influence lignin–enzyme interactions.

### Effects of lignin fractions interaction on enzyme adsorption determined by QCM-D

Since the ethanol-soluble lignin (represented as EL) and residual bulk lignin (represented as MWL) normally aggregate together in real pretreated biomass, enzymatic adsorption on aggregated lignin should be different from adsorption on each individual lignin fraction. Therefore, to better simulate lignin aggregation as it would be in real pretreated biomass, two types of reconstructed lignin films were prepared. EL/MWL-25 film was prepared from a lignin solution containing both EL-25 and MWL-25 in a ratio of 1:1, while EL-MWL-25 film was prepared by coating EL-25 on an existing MWL-25 film.

**Fig. 4:**
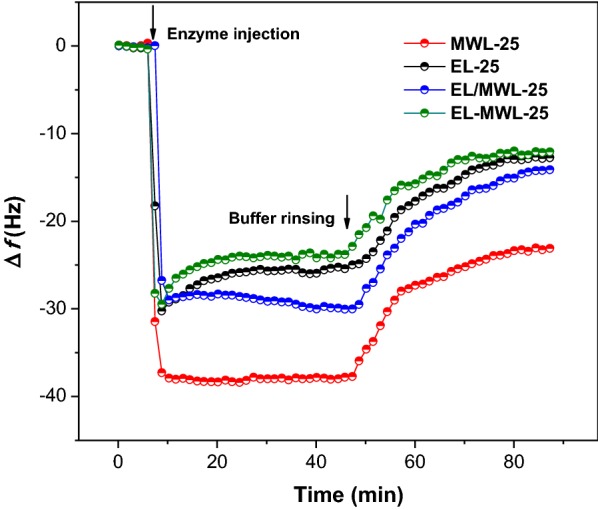
QCM frequency (∆*f*) changes during the enzyme adsorption and desorption on lignin films

Enzyme adsorption and desorption behavior on the lignin films of MWL-25, EL-25, EL/MWL-25, and EL-MWL-25 were compared using QCM-D analysis (Fig. 4). As the enzyme solution was injected, the enzymes were rapidly adsorbed on all the lignin films. Adsorption equilibrium was reached in a short time period for both MWL-25 and EL/MWL-25 films. However, for EL-25 and EL-MWL-25 films, adsorption equilibrium took a relatively longer time to be reached. Specifically, after maximum enzyme adsorption was reached, an obvious increase of ∆*f* from − 30.38 to − 25.87 Hz could be noted for EL-25 film. This suggested that the binding strength between enzyme and EL-25 was relatively weak, leading to enzyme desorption. Similarly, an increase of ∆*f* from − 29.47 to − 24.11 Hz was observed for EL-MWL-25 film. This was due to the coating of EL-25 on MWL-25, which contributed to its similar adsorption behavior with EL-25. As the adsorption equilibrium was reached, the absolute values of *M*_max_ were obtained and were 38.09, 25.87, 28.07, and 24.11 Hz for MWL-25, EL-25, EL/MWL-25, and EL-MWL-25 films, respectively (Table 5). The corresponding maximum adsorption capacities were then calculated as 224.73, 152.63, 165.61, and 142.25 ng/cm^2^. This indicated that MWL-25 had the highest enzyme adsorption, and the presence of EL-25 in EL/MWL-25 and EL-MWL-25 films significantly decreased enzyme adsorption.

Next, enzyme desorption was investigated by injecting fresh buffer instead of the enzyme solution (Fig. 4). As the fresh buffer was injected, an obvious increase of ∆*f* was observed for all lignin films, implying enzyme desorption. When ∆*f* became stabilized, the irreversible enzyme adsorption mass could be calculated. These mass values were found to be 137.35, 75.40, 83.54, and 75.58 ng/cm^2^ for MWL-25, EL-25, EL/MWL-25, and EL-MWL-25 film, respectively (Table 5). This confirmed that the MWL-25, representing the residual bulk lignin, showed the highest adsorption affinity for cellulases. Meanwhile, EL-25, representing the ethanol solubilized lignin generated during pretreatment, showed the lowest adsorption affinity. More importantly, the presence of EL-25 obviously reduced the irreversibly enzyme adsorption on the two reconstructed lignin films by 39.2–45.0%.

**Fig. 5:**
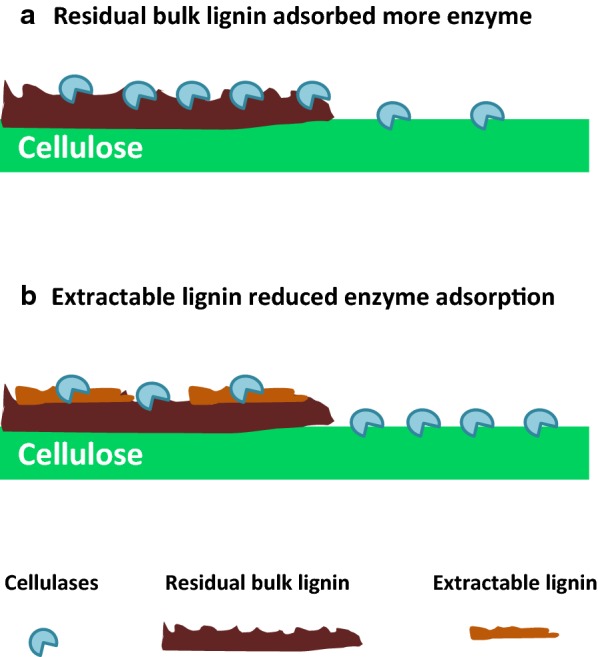
Potential mechanism diagram that EL reduced cellulase adsorption on bulk lignin

Based on the results above, a potential mechanism for the positive effects of EL on enzymatic hydrolysis is illustrated in Fig. 5. The EL with weaker enzyme adsorption affinity might block or shelter the enzyme binding sites on the residual bulk lignin, thus reducing non-productive enzyme binding and therefore enhancing enzymatic hydrolysis of pretreated substrates. Donohoe et al. have proposed that lignin relocalization could enhance enzymatic hydrolysis by increasing the cellulose accessibility [49]; while, our findings reveal another potential mechanism for the positive effects of lignin re-deposition on enzymatic hydrolysis.

## Conclusions

Two lignin fractions representing ethanol-solubilized lignin (EL) and the residual bulk lignin (MWL) were isolated from ethanol organosolv-pretreated substrates. The lignin characterization indicated that compared to MWL, EL was more depolymerized and less condensed. These structural features resulted in lower enzyme adsorption affinity for EL. More importantly, the presence of EL reduced the irreversible enzyme adsorption on reconstructed lignin films. This implied that EL might suppress enzyme non-productive binding on residual bulk lignin in real pretreated substrates, resulting in lignin similar to EL enhancing enzymatic hydrolysis. Understanding the positive effects of EL on enzymatic hydrolysis will help to better design sequential biomass pretreatment and enzymatic hydrolysis processes and lead to a more fruitful bioeconomy.

## Additional files

**Additional file 1: Table S1.** Recovery/removal of all components in ethanol-pretreated materials.

**Additional file 2: Figure S1.** SEM images of (a) EP25, (b) EP25-EW, (c) EP50, and (d) EP50-EW.

## Abbreviations

EL: extractable lignin
MWL: milled wood lignin
QCM-D: quartz crystal microbalance with dissipation
HSQC: heteronuclear single quantum coherence
NMR: nuclear magnetic resonance
S: syringyl
G: guaiacyl
EP: ethanol organosolv-pretreated wood sawdust
HPLC: high-performance liquid chromatography
SEM: scanning electron microscopy
DR: direct red dye
GPC: gel permeation chromatography
*M*_n_: number average molecular weights
*M*_w_: weight average molecular weights
THF: tetrahydrofuran
e-NHI: endo-*N*-hydroxy-5-norbornene-2,3-dicarboximide
TMDP: 2-chloro-4,4,5,5-tetramethyl-1,2,3-dioxaphospholane
AFM: atomic force microscopy
