# Supplementary material for: New strategy to elucidate the positive effects of extractable lignin on enzymatic hydrolysis by quartz crystal microbalance with dissipation
Source: Biotechnol Biofuels. 2019 Mar 19;12:57. doi: 10.1186/s13068-019-1402-2 (PMC6423845; doi:10.1186/s13068-019-1402-2)
Supplement: Supplementary file 1 — Additional file 1: Table S1. Recovery/removal of all components in ethanol-pretreated materials. [file 13068_2019_1402_MOESM1_ESM.docx]

**Additional file 1**

**Table S1 Recovery/removal of all components in ethanol pretreated materials**

| Biomass | Recovery (g)^a^ | | | |  | Removal (%)^b^ | | | |
| --- | --- | --- | --- | --- | --- | --- | --- | --- | --- |
|  | Extractives | AIL ^c^ | Glucan | Hemicellulose |  | Extractive | AIL ^c^ | Glucan | Hemicellulose |
| Raw material | 1.34 | 28.22 | 42.59 | 22.70 |  | - | - | - | - |
| EP25 | 4.64 | 15.25 | 35.88 | 4.00 |  | -246.27 | 45.95 | 84.24 | 82.38 |
| EP25-EW | 0.51 | 15.05 | 35.85 | 4.00 |  | 89.01 | 1.31 | 0.00 | 0.00 |
| EP50 | 4.22 | 11.04 | 36.49 | 5.04 |  | -214.93 | 60.87 | 85.68 | 77.80 |
| EP50-EW | 0.39 | 10.71 | 36.48 | 4.90 |  | 90.76 | 2.95 | 0.03 | 2.78 |

^a^Recovery of each component in pretreated materials is presented in the dry weight, and calculated by mass balance based on 100 g dry raw material. ^b^Removal of each component in EP25 and EP50 is presented in the percentage, and calculated based on raw material; while removal of each component in EP25-EW and EP50-EW is calculated based on EP25 and EP50, respectively. Negative values of extractive removal in EP25 and EP50 indicate the increase in ethanol extractive contents. ^c^AIL refers to the acid insoluble lignin.
