# Supplementary material for: New strategy to elucidate the positive effects of extractable lignin on enzymatic hydrolysis by quartz crystal microbalance with dissipation
Source: Biotechnol Biofuels. 2019 Mar 19;12:57. doi: 10.1186/s13068-019-1402-2 (PMC6423845; doi:10.1186/s13068-019-1402-2)
Supplement: Supplementary file 2 — Additional file 2: Figure S1. SEM images of (a) EP25, (b) EP25-EW, (c) EP50, and (d) EP50-EW. [file 13068_2019_1402_MOESM2_ESM.docx]

**Additional file 2**

**Fig. S1** **SEM images of (a) EP25, (b) EP25-EW, (c) EP50, and (d) EP50-EW.**
